# Evaluation of a questionnaire to assess nutritional knowledge, attitudes and practices in a Thai population

**DOI:** 10.1186/s12937-019-0463-1

**Published:** 2019-07-10

**Authors:** Rungnapa Pongkiatchai, Rewadee Chongsuwat, Nopporn Howteerakul, Patcharanee Pavadhgul, William Ollier, Artitaya Lophatananon

**Affiliations:** 1 Department of Nutrition, Faculty of Public Health, Mahidol University, 420/1 Ratchawithi RD., Ratchathewi District, Bangkok, 10400 Thailand; 2 Departments of Epidemiology, Faculty of Public Health, Mahidol University, 420/1 Ratchawithi RD., Ratchathewi District, Bangkok, Thailand; 3 Division of Population Health, Health Services Research & Primary Care, School of Health Sciences, Faculty of Biology, Medicine and Health, The University of Manchester, Manchester, UK

**Keywords:** Chronic disease, Nutritional knowledge, Nutritional attitude, Nutritional practice, Working age

## Abstract

**Background:**

The rapid increase in non-communicable chronic diseases in people of working age has had a major effect on health care utilization, productivity and economy. Lifestyle and diet are recognized as being major risk determinants involved. Disease prevention strategies need to be based on people’s understanding of nutritional knowledge, attitudes and practice. This study evaluates the validity of a new nutritional knowledge and practice questionnaire specifically developed for assessing individuals of working age in a Thai population.

**Methods:**

The questionnaire was constructed and based on previous relevant literature and its content validity was scrutinized by an expert panel. An exploratory factor analysis (EFA) was performed to reduce the number of questions included. Subsequently, data from a cross-sectional study of 1,032 participants were used to evaluate the reliability and validity of this questionnaire. The validity of the questionnaire constructed for assessing knowledge and attitude was evaluated using Confirmatory Factor Analysis (CFA). For the practice component, set criteria were applied to determine the final variables used.

**Results:**

CFA of the nutritional knowledge component suggested that all the variables in the model fitted with the data (*χ*^2^ = 80.17, *df* = 66, *p* > 0.05, CFI = 0.99, RMSEA = 0.01, SRMR = 0.02). The CFA final model for the nutritional knowledge included three factors (food recommendation, nutrients related to diseases, and healthy diet) with a total of 14 questions. For nutrition attitude, CFA also revealed a good fit (*χ*^2^ = 178.14, *df* = 93, *p* < 0.001, CFI = 0.99, RMSEA = 0.03, SRMR = 0.03). The final CFA model for nutritional attitude included three factors (food choice, healthy diet and food recommendation) with a total of 16 questions. For practice items, the number of questions was reduced from 76 to 60.

**Conclusions:**

Questionnaire development should use a logical, systematic and structured approach. Results from our evaluation process demonstrates the construction validity of the nutritional knowledge and practice questionnaire developed. This questionnaire can be further modified for use in other countries within the region.

**Electronic supplementary material:**

The online version of this article (10.1186/s12937-019-0463-1) contains supplementary material, which is available to authorized users.

## Background

Changes in society and the global economy during the past two to three decades have resulted in significant changes in the nutritional status and health of populations, particularly in developing countries [1]. Thailand is no exception; this country has undergone significant social and economic changes since 1997 [2]. These changes have led to the increasing introduction of a western lifestyle where Thai people consume higher fat foods and “fast-foods” together with an increasing consumption of a less healthy diet and a more sedentary lifestyle [1]. These are major causes of non-communicable diseases (NCDs) such as cardiovascular disease, osteoporosis, and diabetes [3–5]. Thus changes in society have led to subsequent changes in disease prevalence; reciprocally, increases in chronic disease prevalence then changes society further [6].

Level of educational attainment is one of a number of factors that influence nutritional knowledge. Furthermore, nutritional knowledge and attitude are correlated with each other and both play key roles in influencing nutritional behavior [7]. Given a changing and increasing trend of western lifestyle in Thailand, it is important to gain a clear insight into the population-based knowledge of diet and the changing patterns of behavior, both increasing risk of chronic disease development, especially in those of working age [4]. This knowledge will facilitate the introduction of interventions that prevent or reduce future disease development. Epidemiological studies into the relationship between chronic disease development and nutritional risk exposures can only be achieved through the appropriate collection of robust information on the factors that determine our knowledge, attitudes, practices and preferences relating to food and nutrition. Such data can only be generated by the development of appropriate questionnaires and these have to be validated and assessed within the social context of the populations they are designed for. This study evaluates a questionnaire specifically constructed to assess nutritional knowledge, attitude, and practice in a working Thai population.

## Methods

### Study design

This study was conducted using a cross-sectional approach. Study approval was gained from the Ethical Review Committee for Human Research, Faculty of Public Health, Mahidol University, Thailand (COA. No. MUPH 2014–189). Data were collected using the nutritional knowledge and practice self-administered questionnaire for the period from October 2015 to September 2016.

Participants were recruited from the Northern, Southern, Northeastern and Central regions of Thailand between January and September of 2015. The criteria for study inclusion were (i) Thai nationality (ii) living in a selected area for a minimum of 3 months (iii) being aged between 18 and 60 years (iv) ability to read and write Thai (v) being in good health and not affected with diseases such as cancer and cardiovascular disease (vi) giving informed consent.

### Questionnaire development

Prior to this study, a nutritional knowledge and practice questionnaire was developed by combining a trawl of relevant information in peer-reviewed published literature with that gained from discussion with an expert panel. The initial questionnaire list of 131 questions/items aimed at measuring nutritional knowledge, attitude and practice in Thai people working age. The knowledge component comprised multiple-choice questions, each with 4 answers. Correct answers scored one point and incorrect answers zero points. All answers reported as ‘don’t know’ were coded as incorrect. For the “attitude” component a 5-point scale ranging from 1 (strongly disagree) to 5 (strongly agree) was used. A Likert scale was used to quantify the results of “attitude”. For “nutritional practice” (P), the response scale was classified as: seldom eaten (0-1 days/week), often eaten (2–3 days/week) and always eaten (4–7 days/week).

### Validation process

Prior to the validity process, Exploratory Factor Analysis (EFA) was employed to both identify important factors and to selectively reduce the number of items in the questionnaire. The process of questionnaire development is depicted in the flow chart (see Additional file 1: Figure S1). Results from EFA were used to derive a subsequent version of the questionnaire (see Additional file 2: Table S1 and S2). In this article, we present results from data collected using the refined version of the questionnaire after EFA analysis. Data were collected from 1080 subjects residing in the Northern, Northeast, Southern, and Central regions of Thailand. The participants’ weight and height were also measured. Body mass index was computed and classified into 4 categories according to World Health Organization recommendation on body-mass index (BMI) cut-off points for determining overweight and obesity in Asian populations [8, 9]. 48 subjects were excluded due to missing data. Data from 1032 subjects were included in the analysis.

### Data analysis

Descriptive statistics were used to summarize the demographic data. Confirmatory Factor Analysis (CFA) was used to evaluate and confirm the factorial structure of nutritional knowledge and attitude collected using the questionnaire. CFA is a technique used to evaluate the level of fit of the data to the specific, theory-derived measurement model where items contribute load only to the factors they were designed to measure [10]. Furthermore, these factors should be correlated. Finally, errors across variables should be uncorrelated. It is noted that smaller factor loadings result in larger error variances, however this can be improved by re-specification of the model. To estimate the parameters of the model, the model itself must be properly defined. “Goodness-of-fit” measures underlying the analysis of the CFA [11], included chi-square (*X*^*2*^) which shows the difference between observed and expected covariance matrices; values closer to zero indicate a better fit. Researchers may fail to reject an inappropriate model where sample sizes are small and reject an appropriate model where sample sizes are large. Consequently, other measures of ‘goodness of fit’ have been developed. Comparative fit index (CFI) analyzes the model fit by examining the discrepancy between the data and the hypothesized model, while adjusting for the issues of sample size inherent in the chi-squared test of model fit, and the normed fit index. The standardized root-mean-square residual (SRMR) is the square root of the discrepancy between the sample covariance matrix and the model covariance matrix. The root mean square error of approximation (RMSEA) avoids issues due to sample size by analyzing the discrepancy between the hypothesized model, with optimally chosen parameter estimates, and the population covariance matrix. The accepted approach for assessing goodness of fit requires that the chi-square (*X*^*2*^) analysis should have a non-significant *p*-value and a *X*^*2*^/*df* value that is at 3 or below. It is important to consider that the modified *X*^*2*^ from re-specification is usually upwardly biased with sample size [12, 13]. The calculated value for CFI should lie between 0 and 1, with a value over 0.90 indicating a good fit. A value for SRMR should be at 0.08 or below. RMSEA values of approximately 0.06 or below indicates an acceptable fit, with values 0.05 or less, indicating good fit [14]. If the analysis indicated a possible data-model misfit, model modification was applied. This modification involved further re-specification by (i) adding a parameter in the model with a large modification index (MI) and (ii) deleting a parameter from the model with a non-significant path.

For the “practice component”, the items included were based on a food frequency questionnaire (FFQ) [15]. The criteria used to exclude food items included: (i) food items deemed as being seasonal food, (ii) any food item that was consumed in less than 60% of our subjects, (iii) food items that had never been consumed by study subjects. This process was used to reduce and refine of items included.

## Results

### Demographic characteristic

Demographic characteristic of the population sample are summarized in Table 1. 1,032 out of 1,080 participants completed the nutritional knowledge and practice questionnaire, (response rate 95.6%). Most of participants were female (62%), married (59.5%) and had attained an undergraduate university degree or higher (51.2%). The participants’ age ranged from 18 to 62 years with a mean age of 38 years. For BMI, 21.5% of subjects were classified as overweight, 22.3% were class I obese and 2.8% were class II obese.

**Table 1:** General characteristics of participants

Characteristics	Cross-sectional *(n* = 1032)	%
Sex		
Male	397	38.5
Female	635	61.5
Age (years)		
18–32	310	30
33–47	526	51
48–62	196	19
BMI (kg/m^2^)		
Standard (< 23.0)	551	53.4
Overweight (23.0–24.9)	222	21.5
Class I obesity (25.0–29.9)	230	22.3
Class II obesity (≥30.0)	29	2.8
Marital status		
Single	339	32.8
Married	614	59.5
Divorce/separated	79	7.7
Education		
High school and lower	289	28
Diploma / Vocational school	215	20.8
Bachelor degree and higher	528	51.2
Occupation		
Employee	301	29.2
Private company staff	207	20.1
Government staff	276	26.7
Self employed	108	10.5
Agricultures	140	13.6
*Salary (THB)*^a^		
< 15,000	282	27.3
15,000-25,000	547	53
25,001- 35,000	142	13.8
35,001-45,000	42	4.1
45,001-60,000	19	1.9

^a^(1 USD = 35.2 THB)

### CFA results for nutritional knowledge

**Fig. 1:**
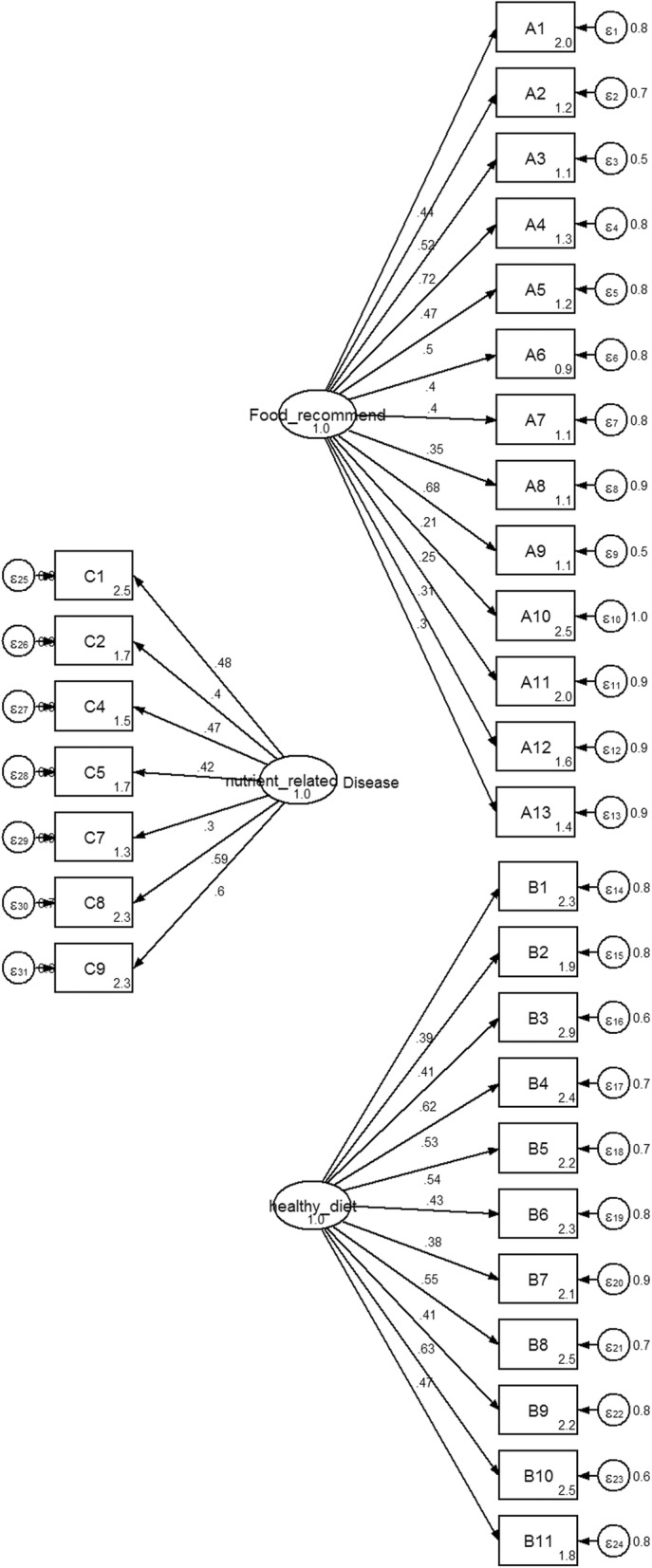
Standardized estimated factor items loading, error variances for nutritional knowledge 3 factors, 31 items Legend: A = basic nutritional knowledge, B = food based dietary guidelines, C = diet related disease knowledge

**Fig. 2:**
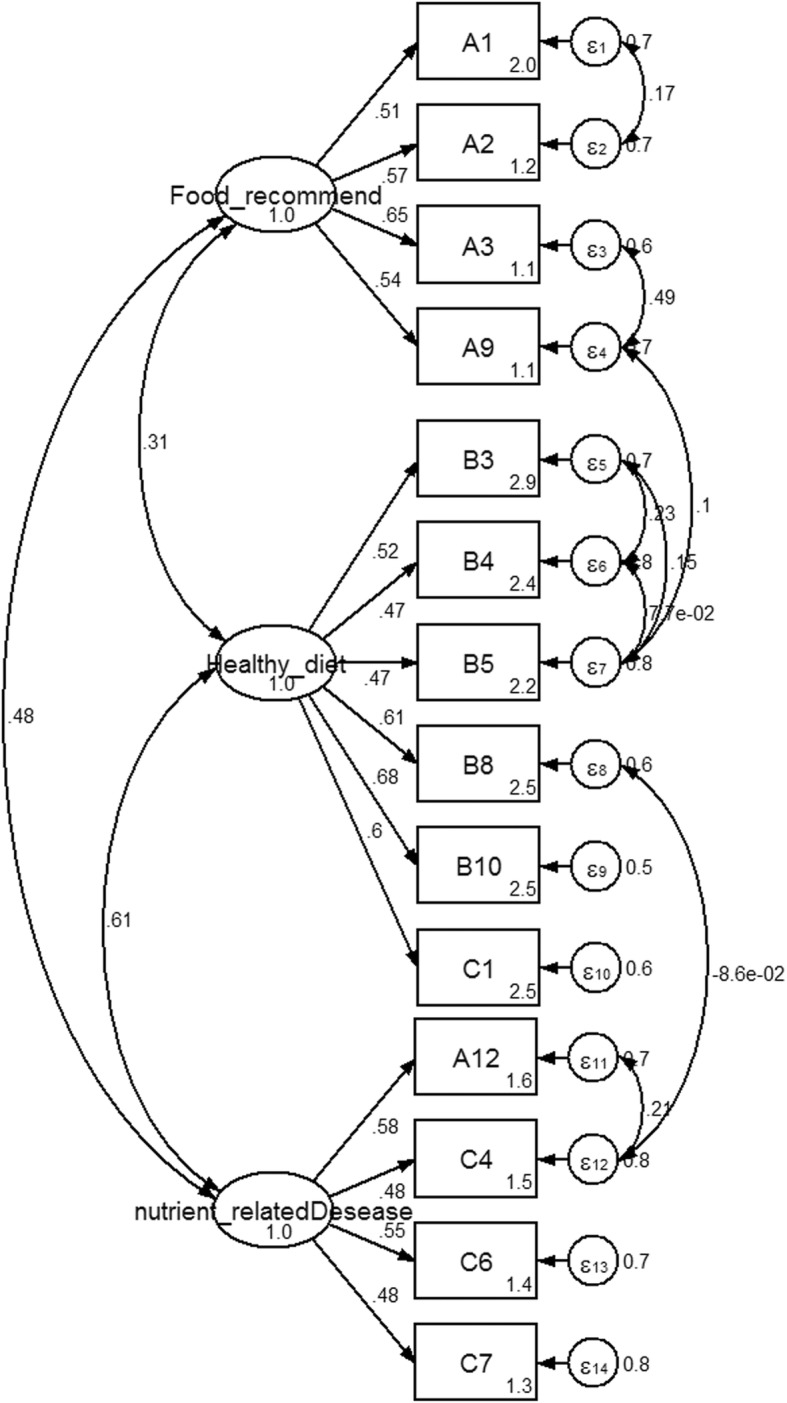
Standardized estimated factor items loading, error-variances and covariance for nutritional knowledge 3 factors, 14 items. Legend: A = basic nutritional knowledge, B = food based dietary guidelines, C = diet related disease knowledge

The initial fitted hypothesized model (3 factors; food recommendation, nutrient related to diseases and healthy diet, with a total of 31 items) suggested a model misfit (*χ*^*2*^)3178.83, df = 434, *p*-value = 0.000, CFI = 0.59, RMSEA = 0.08 and SRMR =0.14 with *χ*^*2*^/df = 7.32, (Fig. 1). The modification indices (MIs) showed the expected parameter change statistics for each item. The model was re-specified and eventually 9 items with a low factor item loading from three factors were excluded. To ensure the model fitted well, covariance values were also added. The final CFA model for “nutritional knowledge” was composed of three factors (food recommendation, nutrient related to diseases and healthy diet) with a total of 14 items. Parameter estimation results after model modification revealed a good fit for the model as indicated by a reduced chi-square value (from 465.5 to 80.2 with 66 degrees of freedom, *p*-value> 0.05). The value of CFI, RMSEA and SRMR were 0.99, 0.01 and 0.02 respectively, indicating a large improvement in the model fit. The parameter estimates of the CFA are shown in Fig. 2.

### CFA results for nutritional attitude

**Fig. 3:**
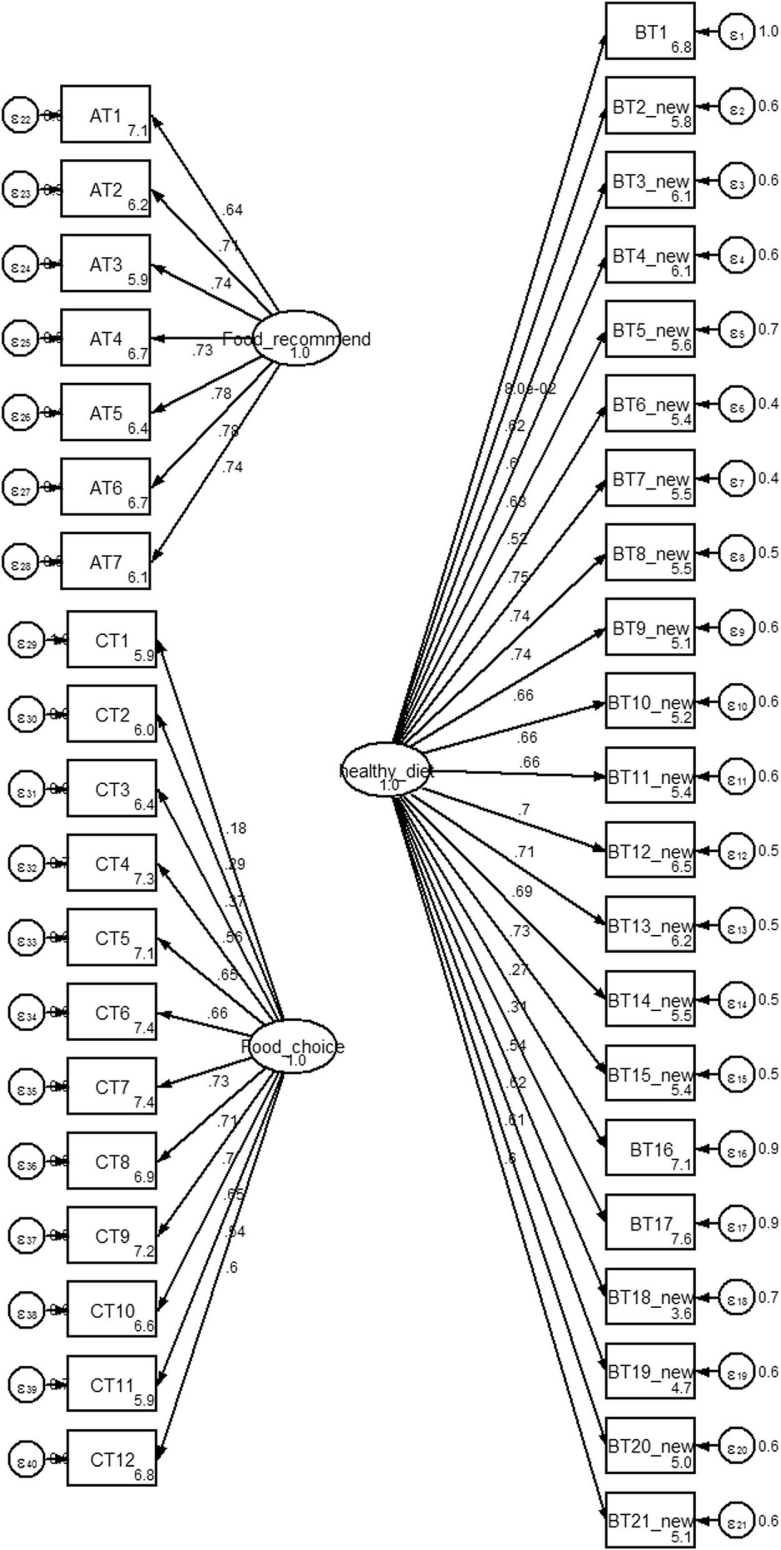
Standardized estimated factor items loading, error variances for nutritional attitude 3 factors, 40 items. Legend: AT = food based dietary guideline attitude, BT = balance diet and their variety attitude, CT = food choice attitude

**Fig. 4:**
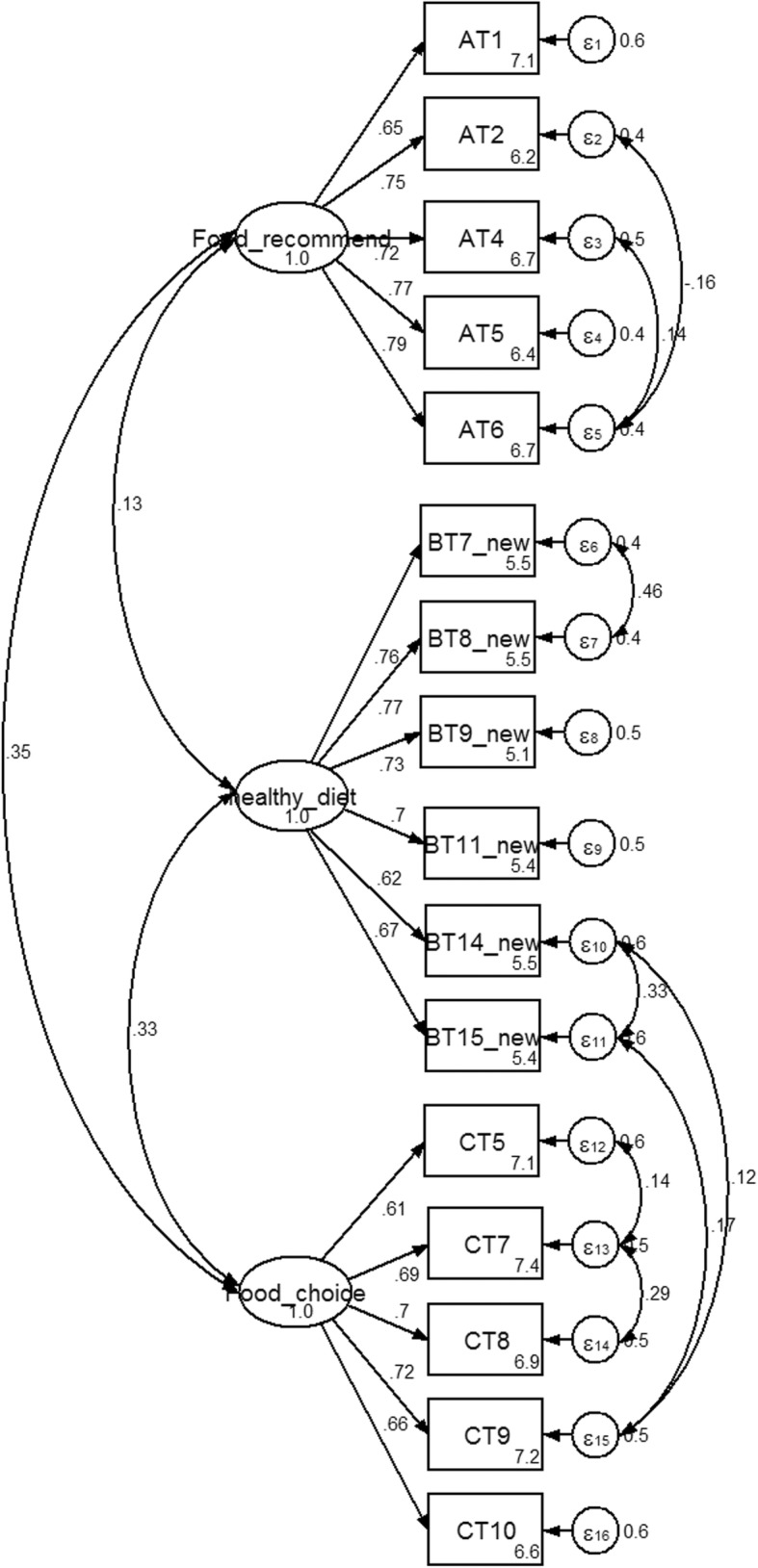
Standardized estimated factor items loading, error-variances and covariance for nutritional attitude 3 factors, 16 items. Legend: AT = food based dietary guideline attitude, BT = balance diet and their variety attitude, CT = food choice attitude

The first hypothesized model consisted of 3 factors (food choice, healthy diet and food recommendation with a total of 40 items), revealed a ***χ***^***2***^ value of 5503.0, df = 740, p-value = 0.000, CFI = 0.75, RMSEA = 0.08 and SRMR =0.11 with ***χ***^***2***^/df = 7.4 (Fig. 3). The initial model did not fit the sample data. Fifteen items were excluded due to low factor item loading. Any non-significant paths from the three factors were also deleted. Covariance values were added. This procedure significantly improved the model. The final CFA model for the nutritional attitude was composed of three factors (food choice, healthy diet and food recommendation with a total of 16 items). The Chi-square value was reduced from 801.1 to 178.1 with 93 degrees of freedom. The three factor model failed to achieve an exact fit (***χ***^***2***^/df = 1.9, *p* < 0.05). An acceptable fit was indicated by CFI, RMSEA and SRMR values (0.99, 0.03 and 0.03, respectively) [16]. All of the standardized factor loadings were greater than 0.4. The parameter estimates of the CFA are shown in Fig. 4.

### The results of nutritional practice (FFQ)

The process of model refinement reduced the food items (practice) from the original 76 to 60 items, including 6 items on milk and dairy products, 7 items on rice, 10 items on meat and products, 4 items on vegetables, 4 items on fruits, 5 items on cereal, 2 items on dessert, 6 items on beverages, 3 items on fats, 13 items on miscellaneous (Table 2).

**Table 2:** Food frequency questionnaire with 60 items

Food groups	Food items
Milk and dairy products (6 items)	Whole milk, fermented milk, low fat milk, sweetened whole milk, yoghurt, cheese etc.
Rice (7 items)	Steamed rice, glutinous rice, noodle, instant noodle etc.
Meat and products (10 items)	Pork, chicken, beef, fish, egg, bacon etc.
Vegetables (4 items)	Chinese kale, bitter cucumber, carrot, sesbania flower
Fruits (4 items)	Banana, mango, orange, guava
Cereals and products(5 items)	Peanut, mung bean, soy milk, tofu, sunflower seed
Dessert (2 item)	Any dishes cooked with coconut milk (such as banana with coconut milk), any dishes cooked with syrup (such as black grass jelly with syrup)
Beverages (6 items)	Fruit juice (40%), soda beverage, cold coffee, green tea frappe, fruit frappe
Fats (3 items)	Animal oil, rice barn oil, soy bean oil
Miscellaneous (13 items)	Hamburger, bakery, bread, cake, green curry etc.

## Discussion

Previous studies both in Europe and China have assessed nutritional knowledge, attitude and eating behavior using local inventory questionnaires [17, 18]. These studies have focused mainly on either the nutritional knowledge, attitudes and practices of undergraduate students or of elderly populations. In Thailand, previous studies have developed nutritional knowledge attitude and practice questionnaires for specific age groups such as children and the elderly [19, 20] however none has reported instrument validation process.

This study has developed and evaluated the construct validity of the Thai adult nutritional knowledge, attitude and practice questionnaire for just such purposes. Measuring nutritional knowledge, attitude and practice in adults is challenging as an appropriate questionnaire has not been available. Other research instruments that exist are well established but are unlikely to be appropriate for Thais [21–23]. The nutritional knowledge attitude and practice questionnaire has social and cultural features that should be considered in a number of dimensions. To obtain information on nutritional behavior in any population, the instrument should include questions related to knowledge and attitudes related to food consumption and practice [24].

Confirmatory factor analysis can be used to verify the structure of a set of observed variables in order to determine whether the extracted items show acceptable fit for the data collected. Cut-off points of model fit criteria can be set and used to determine acceptable values for the model of nutritional knowledge [25–27]. CFA is often used to confirm hypotheses and uses pathway analysis diagrams to represent variables and factors [28]. In this study, the CFA results helped evaluate the nutritional attitude structure of the questionnaire. The model showed an acceptable fit with indices close to the nominal value. The CFA results for attitude, however, initially failed to fit the collected data as demonstrated by the highly significant Chi-square test for goodness of fit. It has been suggested that this test is overly sensitive to sample size [13, 25, 26]. The highly significant goodness of fit test may have been affected by the external factors such as sample size, the number of parameters and the degrees of freedom to sample size ratio [13, 29]. Nevertheless, the other indices suggested an acceptable fit. It was concluded that the nutritional knowledge and attitude components of the questionnaire yielded logical construct validity.

With regard to nutritional practice, we applied the food frequency questionnaire to evaluate eating behavior. The food items reported in the FFQ were covered by five food groups and dietary guidelines for Thais [30]. We applied 3 criteria to reduce the number of items. The remaining items are those normally consumed by Thai people.

Our current study still has some limitations as we did not stratify our analysis by gender as a variable. Males and females may potentially display different knowledge and attitudes to nutrition. Future studies should investigate this in Thai adults using the nutritional knowledge and practice questionnaire. Furthermore the age range for the adult working population is relatively broad and within this range some variation on attitude and knowledge relating to nutrition may also exist.

## Conclusion

This study demonstrates that final version of the questionnaire has acceptable levels of constructed validity and can be used to assess nutritional knowledge, attitude and practice in a general adult working population of Thais. This questionnaire can be further modified for use in neighboring countries in the region that share a similar culture.

This questionnaire can also be used to identify gaps in the public’s nutritional knowledge and to evaluate the success of public health education campaigns and nutritional interventions. It can also identify nutritional knowledge and practice determinants associated with diseases risk.

## Additional files

Additional file 1: **Figure S1.** Flow chart describing the development and validation of the NKAP 1 questionnaire. (PDF 13 kb)

Additional file 2: **Table S1.** Results of the EFA of the nutritional knowledge component in validation study (*n* = 103)*.**Tables S2.** Results of EFA of the nutritional attitude component in validation study (*n* = 103). (PDF 53 kb)

## Data Availability

The datasets used and/or analyzed during the current study available from the corresponding author on reasonable request.

## Abbreviations

CFA: Confirmatory Factor Analysis
CFI: Comparative Fit Index
df: Degree of freedom
EFA: Exploratory Factor Analysis
FFQ: Food Frequency questionnaire
RMSEA: Root Mean Square Error of Approximation
SRMR: Standardized Root-Mean-square Residual

## References

[1] Ministry of Public Health (2008). Thailand Health Profile Report 2008-2010.

[2] Ministry of Public Health (2008). Thailand Health Profile Report 2008-2010.

[3] Aekplakorn W. Thai National Health Examination Survey, NHES V. Nonthaburi:. The Graphico system co.Ltd.; 2014.

[4] Health Information System Development Office. Thailand Health Profile 2008-2010. 2010. https://www.hiso.or.th/hiso5/report/report9.php. Accessed 10 July 2017.

[5] Murakami K, Okubo H, Sasaki S (2005). Effect of dietary factors on incidence of type 2 diabetes: a systematic review of cohort studies. J Nutr Sci Vitaminol (Tokyo).

[6] Kosulwat V (2002). The nutrition and health transition in Thailand. Public Health Nutr.

[7] Azizi M, Aghaee N, Ebrahimi M, Ranjbar K (2011). Nutrition knowledge, the attitude and practices of college students. Facta Universitatis.

[8] Expert Consultation WHO (2004). Appropriate body-mass index for Asian populations and its implications for policy and intervention strategies. Lancet.

[9] Jitnarin N, Kosulwat V, Rojroongwasinkul N, Boonpraderm A, Haddock CK, Poston WS (2011). Prevalence of overweight and obesity in Thai population: results of the National Thai Food Consumption Survey. Eat Weight Disord.

[10] Mueller RO, Hancock GR, Smelser NJ, Baltes PB (2001). Factor analysis and latent structure, confirmatory. International encyclopedia of the Social & Behavioral Sciences.

[11] Brown TA, Kenny DA (2006). Confirmatory factor analysis for applied research.

[12] Hooper D, Coughlan J, Mullen MR (2008). Structural equation modelling: guidelines for determining model fit. Electron Bus Res Methods.

[13] McCoach DB, Gable RK, Madura JP (2013). Instrument development in the affective domain.

[14] Fonseca M (2013). Principles and practice of structural equation modeling, third edition by rex B. Kline. Int Stat Rev.

[15] Pongkiatchai R. A study of nutrient intake of high school students in Bangkok using semiquantitative food frequency questionnaire Bangkok. Bangkok: Mahidol University; 1999.

[16] Chen X, Hu Y, Zhu D, Li J, Zhou L (2016). Chinese version of the aging perceptions questionnaire (C-APQ): assessment of reliability and validity. Aging Ment Health.

[17] Parmenter K, Wardle J (1999). Development of a general nutrition knowledge questionnaire for adults. Eur J Clin Nutr.

[18] Marietta AB, Welshimer KJ, Anderson SL (1999). Knowledge, attitudes, and behaviors of college students regarding the 1990 nutrition labeling education act food labels. J Am Diet Assoc.

[19] Polsiri A (2008). Knowledge, attitudes and behaviors about food consumption of Ramkhamhaeng University undergraduate students. Ramkhamhaeng J.

[20] Piaseu N, Tatsanachantatanee D, Kittipoom S, Putwatana P (2009). Nutritional knowledge, attitude toward food, food behavior, and nutritional status among nursing students. Rama Nurs J.

[21] Feren Anne, Torheim LivE, Lillegaard IngerT L (2011). Development of a nutrition knowledge questionnaire for obese adults. Food & Nutrition Research.

[22] Hendrie GA, Cox DN, Coveney J (2008). Validation of the general nutrition knowledge questionnaire in an Australian community sample. Nutr Diet.

[23] Medeiros LC, Hillers VN, Chen G, Bergmann V, Kendall P, Schroeder M (2004). Design and development of food safety knowledge and attitude scales for consumer food safety education. J Am Diet Assoc.

[24] Green EC (2001). Can qualitative research produce reliable quantitative findings?. Field Methods.

[25] Hair JF, Black WC, Babin BJ, Anderson RE (2010). Multivariate data analysis: a global perspective.

[26] Kline RB (2015). Principles and practice of structural equation modeling.

[27] Lim T-P, Chye FY, Sulaiman MR, Suki NM, Lee J-S (2016). A structural modeling on food safety knowledge, attitude, and behaviour among Bum Bum Island community of Semporna, Sabah. Food Control.

[28] Yong AG, Pearce S (2013). A Beginner’s guide to factor analysis: focusing on exploratory factor analysis. Tutor Quant Methods Psychol.

[29] Marsh HW, Balla J (1994). Goodness of fit in confirmatory factor analysis: the effects of sample size and model parsimony. Qual Quant.

[30] Bureau of Nutrition. Food-based dietary guidelines for Thai: Bangkok Ministry of Public Health; 2001. http://www.fao.org/3/a-as887e.pdf. Accessed 28 June 2016.

