# Supplementary material for: Evaluation of a questionnaire to assess nutritional knowledge, attitudes and practices in a Thai population
Source: Nutr J. 2019 Jul 10;18:35. doi: 10.1186/s12937-019-0463-1 (PMC6621999; doi:10.1186/s12937-019-0463-1)
Supplement: Supplementary file 1 — Figure S1. Flow chart describing the development and validation of the NKAP 1 questionnaire. (PDF 13 kb) [file 12937_2019_463_MOESM1_ESM.pdf]

Supplement figure1. Flow chart describing the development and validation of the NKAP questionnaire

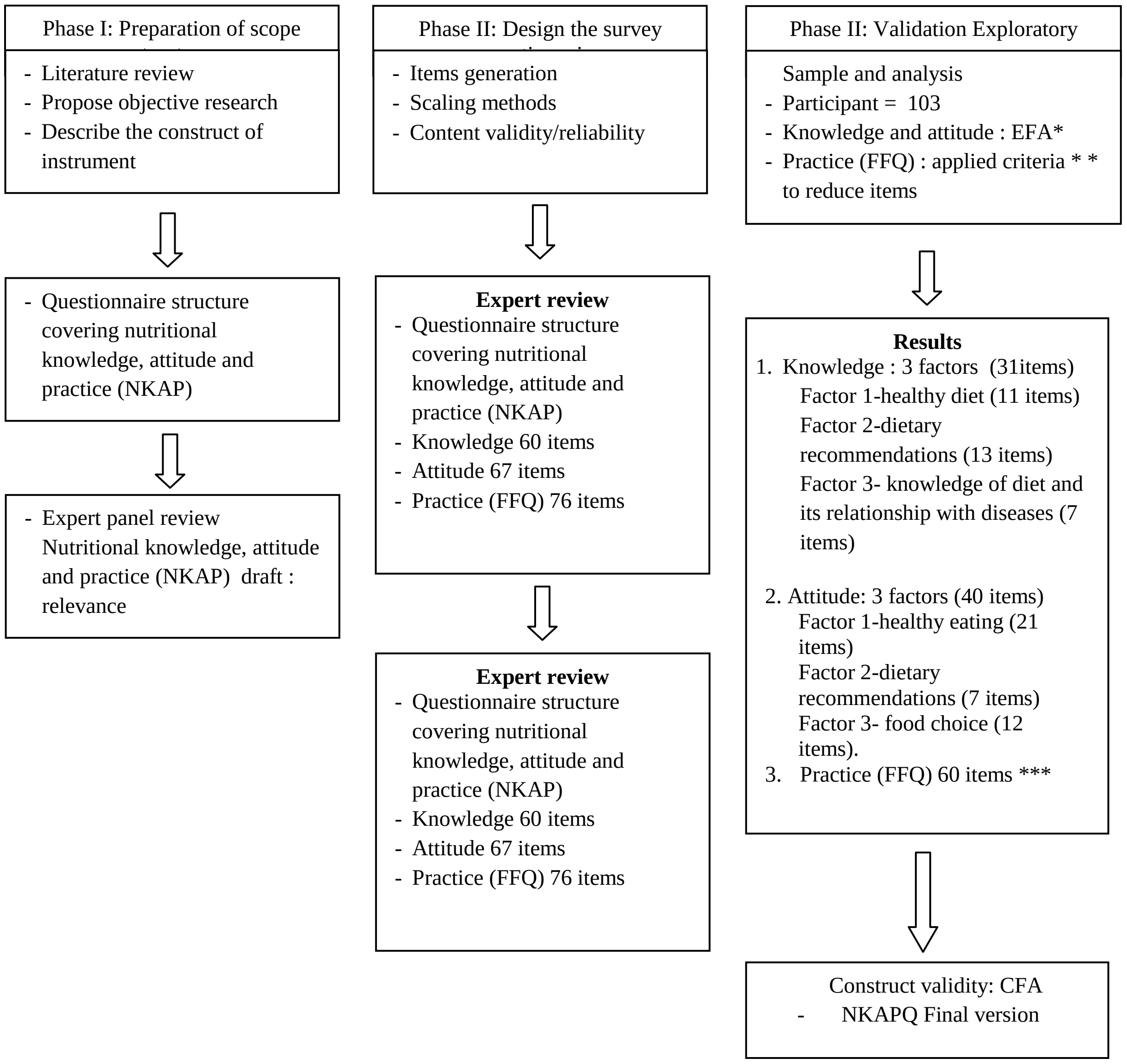

* The results of EFA showed in Supplement1and Supplement table2

** 1) food items that are deemed as seasonal food;
2) Any food items that were consumed in less than 60% of our subjects
3) Food items that never been consumed by study subjects.
