# Supplementary material for: Evaluation of a questionnaire to assess nutritional knowledge, attitudes and practices in a Thai population
Source: Nutr J. 2019 Jul 10;18:35. doi: 10.1186/s12937-019-0463-1 (PMC6621999; doi:10.1186/s12937-019-0463-1)
Supplement: Supplementary file 2 — Table S1. Results of the EFA of the nutritional knowledge component in validation study (n = 103)*.Tables S2. Results of EFA of the nutritional attitude component in validation study (n = 103). (PDF 53 kb) [file 12937_2019_463_MOESM2_ESM.pdf]

Supplement table 1 Results of the EFA of the nutritional knowledge component in validation study (n=103)*

Items	Factors loading		
	1	2	3
**Dietary recommendation**			
K1: If you were trying to cut down meat, which group of foods should you eat instead of meat	.705		
K2: Which foods contain a lot of calcium	.540		
K3: Which one of the following statements is the key to a healthy way of eating	.746		
K4: Which of the following statement can decrease weight gain/overweight	.352		
K5: Which of the following statements do experts say people should eat regularly regarding the amount of vegetables and fruits regularly	.519		
K6: Which of the following foods would help people to avoid becoming fat	.416		
K7: Which of the following behaviors can cause cross-contamination of foods	.411		
K8: If a person wanted to reduce the amount of fat in their diet, which would be the best choice	.634		
K9: Which would be the best choice for increasing high antioxidants	.464		
K10: Which of the following foods can increase weight gain/overweight	.850		
K11: What is an indicator to measure overweight/obesity	.768		
**Healthy diet**			
K12: Saturated fats are mainly found in		.573	
K13: A type of oil which contains mostly monounsaturated fat is		.630	
K14: Which of the following is considered good fat in a diet		.613	
K15: Which of the following foods has the highest cholesterol level		.426	
K16: Which of the following statement is true for cholesterol		.473	
K17: Which foods contain a lot of trans fatty acids		.586	
K18: Which of the following statement is true for trans fatty acids		.639	
K19: According to the Thailand Nutrition Flag, how many servings of rice should a person of your age and gender eat each day for good health		.397	
K20: According to the Thailand Nutrition Flag, how many serving of meat should a person of your age and gender eat each day for good health		.382	
K21: What fatty acid do experts say people should avoid		.631	
K22: Which of the following foods has the lowest fat		.405	
K23: How many calories (Dietary Reference Intakes for Thais 2003: DRIs) should a working group of your age and gender consume per day		.444	
K24: A type of food which contains mostly sodium is		.437	
**Knowledge of diet and its relationship with diseases**			
K25: Which kind of foods do you think increase osteoporosis, if a person eats a large amount every day			.246
K26: Which foods contain a lot of fibre			.576
K27: Which foods contain a lot of sodium			.355
K28 Which of the following is true for high sodium intake			.509
K29: Which foods contain a lot of fat			.420
K30: What are the major health problems or diseases that are related to a adding too much salt			.795
K31: which are the healthy foods			.691

*Explore Factor Analysis (EFA) was used to explore and summarize the underlying structure of data set. The assumptions underlying multivariate analysis for the EFA, including normality and no outliers were tested before further analysis was conducted. The results of EFA (nutrition knowledge and attitude) showed in supplement table 1and supplement table 2.

Supplement table 2 Results of EFA of the nutritional attitude component in validation study (n=103)

Items	Factor loading		
	1	2	3
**Dietary recommendation**			
AT1: Thai food guide (food based dietary guidelines and nutritional flag) is a useful tool for planning my food choice.		.797	
AT2: Nutritional flag symbolized nutrition information is easy to understand.		.843	
AT3: I am often aware of the nutrition flag.		.690	
AT4: Nutritional flag is a useful tool for planning a healthy diet.		.675	
AT5: The nutrition information in food based dietary guidelines is easy to understand.		.835	
AT6: Food based dietary guidelines are a useful tool for planning a healthy diet.		.857	
AT7: I am often aware of the food based dietary guidelines.		.793	
**Healthy eating**			
AT8: Eating many different kinds of foods is healthier than eating only a few kind foods.	.457		
AT9: I believe that having fast food every day is healthy.	.586		
AT10: Rice, noodles and bread are a better diet than meat and vegetables.	.619		
AT11: Canned fruit has the same health as fresh fruits.	.685		
AT12: Fruit juice is just as healthy as water to drink.	.589		
AT13: I should eat a lot of sugar so I can have energy.	.660		
AT14: Salt should be added to all foods.	.821		
AT15: I think that I should add extra salt/sugar/fish sauce to my cooked foods.	.708		
AT16: It is impossible to get all the vitamins and minerals I need from food.	.545		
AT17: I need to take vitamin and mineral pills every day.	.556		
AT18: Drinking coffee or green tea /day will give me antioxidants.	.309		
AT19: I do not need to wash my vegetables before I cook them.	.532		
AT20: I believe that adding lime, salt and chili to fresh meat makes it safe to eat without cooking the meat.	.612		
AT21: I like eating white rice more than brown rice.	.642		
AT22: If I eat sugary fruits like durian or tamarind I will not gain weight.	.439		
AT23: It is not healthy to eat a high fat diet.	.485		
AT24: I can have healthy body weight if I eat healthy diet and exercise.	.487		
AT25: I choose food based on taste rather than nutritional value.	.434		
AT26: Foods need to have flavor enhancers, seasoning, or sauces added so they taste good.	.642		
AT27: Add salt/sugar/fish sauce to my food does no harm to my health.	.723		
AT28: Eating a lot of high heat-grilled meat every day is not harmful on my body.	.566		
**Food choice**			
AT29: Skim milk is healthier than semi-skim milk and full fat milk.			.407
AT30: Western foods are always high in fat.			.411
AT31: It is good to eat fresh fruits and vegetables because they are low calorie foods.			.560
AT32: I eat a lot of fresh fruit every day because they have vitamins, minerals and antioxidants.			.436
AT33: My body only needs a little bit of salt to be healthy.			.395
AT34: People who want to lose weight should increase their physical activity			.647
AT35: Eating food with a lot of carbohydrates (such as rice, noodles and bread) makes people gain weight.			.559
AT36: Drinking a lot of sugary drinks can make me gain weight.			.512
AT37: Fresh fruits have benefit more than sugary foods.			.696
AT38: I think I choose Thai foods over other food options.			.289
AT39: Using food labels to choose foods is better than just relying on my own knowledge about what is in the foods.			.444
AT40: I believe that I read food labels before buying foods.			.377
